# Hierarchical Phased-Array Antennas Coupled to Al KIDs: A Scalable Architecture for Multi-band Millimeter/Submillimeter Focal Planes

**DOI:** 10.1007/s10909-024-03110-4

**Published:** 2024-06-17

**Authors:** Jean-Marc Martin, Junhan Kim, Fabien Defrance, Shibo Shu, Andrew D. Beyer, Peter K. Day, Jack Sayers, Sunil R. Golwala

**Affiliations:** 1 California Institute of Technology, 1200 E. California Blvd., Pasadena, 91125 CA USA; 2 Jet Propulsion Laboratory, California Institute of Technology, Pasadena, 91109 CA USA

**Keywords:** KID, Hierarchical antenna, Millimeter wavelength, Submillimeter wavelength, Beam maps, FTS

## Abstract

We present the optical characterization of two-scale hierarchical phased-array antenna kinetic inductance detectors (KIDs) for millimeter/submillimeter wavelengths. Our KIDs have a lumped-element architecture with parallel plate capacitors and aluminum inductors. The incoming light is received with a hierarchical phased array of slot dipole antennas, split into 4 frequency bands (between 125 GHz and 365 GHz) with on-chip lumped-element band-pass filters, and routed to different KIDs using microstriplines. Individual pixels detect light for the 3 higher-frequency bands (190–365 GHz), and the signals from four individual pixels are coherently summed to create a larger pixel detecting light for the lowest frequency band (125–175 GHz). The spectral response of the band-pass filters was measured using Fourier transform spectroscopy (FTS), the far-field beam pattern of the phased-array antennas was obtained using an infrared source mounted on a 2-axis translating stage, and the optical efficiency of the KIDs was characterized by observing loads at 294 K and 77 K. We report on the results of these three measurements.

## Introduction

Future large-aperture (30–50 m) millimeter/submillimeter ground-based telescopes (e.g., CSST, CMB-HB, AtLAST) will require detectors able to observe several spectral bands within the range

75–415 GHz in order to study cold, dusty sources, millimeter/submillimeter time-domain sources, the circumgalactic medium and galaxy cluster intracluster medium using the Sunyaev–Zel’dóvič effect, and the cosmic microwave background intensity and polarization on sub-arcminute scales. Given the cost of such large apertures, it is most efficient if each pixel in the focal plane can sense multiple spectral bands. It is optimal to match the pixel size to the scaling of the diffraction spot size with frequency to avoid oversampling (excessive detector count) or undersampling (loss of angular resolution) the focal plane. Hierarchical antennas, first proposed in [1, 2] and first demonstrated by [3], can meet this need by coherently summing signals from individual pixels to provide this frequency scaling of pixel size, as first noted in [4, 5].

**Fig. 1:**
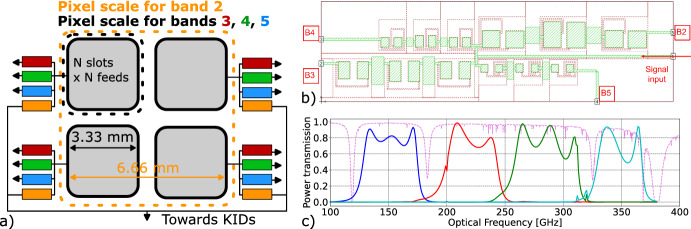
**a** Schematic of the hierarchical phased-array antenna. Each sub-element is similar to the antenna described in [9], though the SiN_x_ dielectric has been replaced by a-Si:H and the shunt capacitor and microstripline dimensions adjusted for the differing dielectric constant. **b** Schematic of the band-pass filter bank [7], with the four bands indicated. **c** Sonnet simulations of the band-pass filter bank with typical atmospheric transmission (pink) overlaid generated from the ATM model with $\text {PWV}=0.5$ at Llano de Chajnantor Observatory. The bands are B2, B3, B4, and B5 in order of increasing frequency

We first introduced the concept for a three-scale hierarchical phased-array slot dipole antenna coupled to TiN_x_ KIDs, covering 75–415 GHz, in [5], and we designed and fabricated a two-scale version with six bands over this frequency range. We found it difficult to model the observed response of these KIDs because TiN_x_ deviates from Mattis–Bardeen theory [6], so we revised the design to use Al KIDs. We also discovered issues with our initial antenna and band-pass filter design that led us to reduce scope to four bands for an initial demonstration (without reducing the antenna’s intrinsic bandwidth). We reported on the revised design, including preliminary optical efficiency and noise measurements, in Shu et al. [7]. Figure 1 shows the two-scale, four-band design. The four frequency bands correspond to the bands 2–5 of the initial 6-band design and are designated as B2, B3, B4, and B5 in the article. The designed mean frequency ($\nu _\text {mean}$) and effective bandwidth ($\Delta \nu _\text {eff.}$) of these bands are listed in Table 1. The use of amorphous hydrogenated silicon (a-Si:H) with a very low loss tangent (close to $10^{-5}$ at RF frequencies and expected to be $\lesssim 10^{-4}$ at millimeter/submillimeter wavelengths) in the microstripline ensures the additional length required for hierarchical summing contributes negligible additional loss.

In this paper, we present the phased-array beam pattern characterization for the four frequency bands, the spectral response of the filter bank measured using Fourier transform spectroscopy (FTS), and the optical efficiency of the KIDs. The beam maps and FTS measurements were obtained using the GPU-accelerated RF readout developed in Minutolo et al. [8]. The devices are operated in a cryostat that provides a 244 mK operating temperature^1^ and is configured with optical windows, so response can be measured up to 40^∘^ off axis.

## Beam Maps

To measure the beam profile of the hierarchical phased-array antenna pixels in each band, we measured the response of the KIDs while scanning a blackbody source, chopped at 8 Hz, mounted on a motorized 2-axis linear translation stage at $\simeq 189~\text {mm}$

**Fig. 2:**
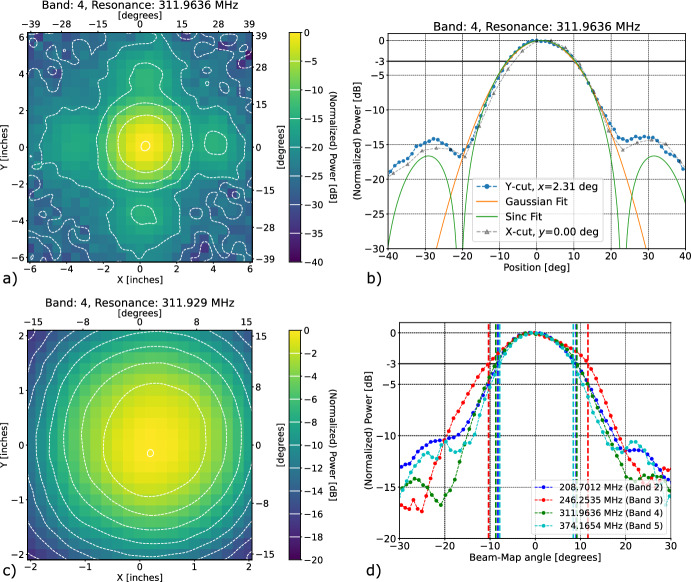
**a** Typical two-dimensional beam map for B4. **b** B4 beam map cross sections at $x=2.31$° and $y=0$. **c** Close-up on central lobe for B4 beam map. **d** Beam cross sections for all bands. The vertical lines indicate the FWHMs, reported in Table 1. The similarity of the B2 and B4 beams demonstrates the hierarchical summing is functional

**Fig. 3:**
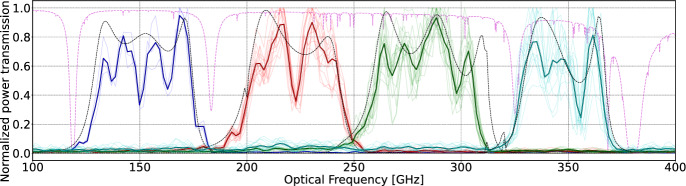
Measured power transmission spectrum. The thin solid lines show the spectral responses of individual KIDs, while the thick solid lines show the mean response for each band. The dashed black lines show the same Sonnet simulations and the dashed pink line the same atmospheric transmission as in Fig. 1

distance from the KIDs. The 300-mm travel of the beam mapper enables measurement of the first beam side lobe. We measure the amplitude of the resulting modulation in the RF network transmission $S_{21}(f_{\textrm{res}})$, which is proportional to the KID frequency shift and thus the intensity of light received. Figure 2 shows example beam maps. Because the antenna illumination pattern in the focal plane is approximately uniform over a square 3.3 mm on a side, we expect the beams to be sinc functions. Figure 2 compares cross-sectional cuts to the expected beam shape and a phenomenological Gaussian fit to the main lobe. The model reproduces qualitatively the expected side lobe. Most importantly, B2’s FWHM and side lobe positions match the expectation from hierarchical summing (pixel size twice as large) and are narrower/closer in than B3’s. Table 1 shows the measured FWHMs. Further modeling will be done to better understand the discrepancy between observed and expected side lobe positions, the filling-in of the sinc function’s first null, and the somewhat elevated shoulder seen in B2’s beam.

## Band-Pass Measurements

We use a Martin–Puplett interferometer to do Fourier transform spectroscopy to measure spectral response. The interferometer is fed by a 1050 *C* cavity blackbody, chopped at 8 Hz. We again monitor the KIDs’ modulated response. The resulting interferogram is Fourier-transformed to generate the power transmission spectrum/response of the whole system (cryostat window, optical filters, hierarchical antenna, band-pass filters, microstripline, and KIDs) between 100 and 400 GHz.

Figure 3 shows the measured spectral responses, both for each KID and averaged over each band, along with the Sonnet simulation of the band-pass filters. (We expect the response of all other elements to be smooth functions of frequency, nearly flat over any individual band.) Table 1 shows the effective bandwidth and band center of each band, obtained via $\Delta \nu _\text {eff.} = \int t_\text {norm}(\nu )\, \textrm{d}\nu$ and $\nu _\text {mean}=\int t_\text {norm}(\nu )\,\nu \, \textrm{d}\nu / \Delta \nu _\text {eff.}$, with $t_\text {norm}(\nu )$ the normalized power transmission. The results differ from the Sonnet simulation by 14–20% for $\Delta \nu _\text {eff.}$ and 1–5% for $\nu _\text {mean}$, where the former can be attributed primarily to in-band transmission variation rather than the position of the band edges (with the exception of B4). The internal minima in the bands are yet to be investigated on whether they are due to the free-space optical path or from the detector transmission lines.

**Fig. 4:**
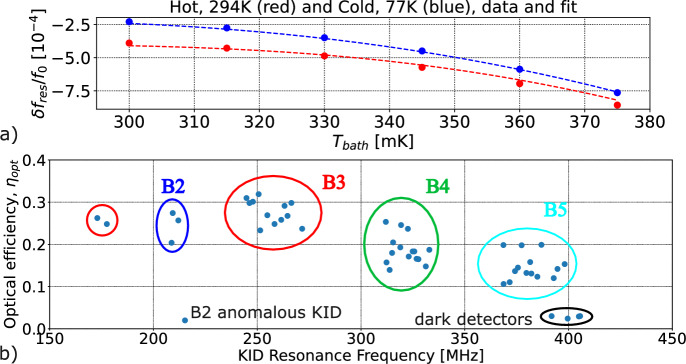
**a** Data points (dots) and corresponding fits (dashed lines) of $\delta f_{\textrm{res}} / f_0$ variation as a function of $T_\text {bath}$ for $T_\text {load} = 77$ K (blue) and $T_\text {load} = 294$ K (red). **b** Fitted optical efficiency ($\eta _{\textrm{opt}}$) for each KID. Colored circles indicate the spectral band of each KID. The anomalous KID is probably not well coupled to its antenna due to a fabrication flaw. The “dark detectors” are intentionally not coupled to antennas (Color figure online)

## Optical Efficiency

To measure the end-to-end optical efficiency, we place a warm ($T_{\text {load}} = 294$ K) or cold ($T_{\text {load}} = 77$ K) blackbody load^2^ in front of the vacuum window, terminating the entire beam that exits the cryostat, and measure the change in KID resonant frequency, repeating the measurement at bath temperatures $T_\text {bath}$ between 300 and 375 mK to break model degeneracies. A super air knife (Exair) generates a laminar air flow parallel to the cryostat window to prevent water vapor from condensing on the window while observing the 77 K blackbody. We fit the data to Eq. 1 (e.g., [10]) to measure $\eta _{\textrm{opt}}$ for each KID:

1 $$\begin{aligned} \frac{\delta f_\text {res}}{f_0}&= -\frac{\alpha }{2} \kappa _2\left( \left[ \frac{\eta _{\textrm{opt}} \eta _{pb} k_\textrm{B} \Delta \nu _\text {eff.} (T_{\text {load}}+T_\text {exc})}{R V \Delta _0} + n_{\text {th}}^2 + \frac{1}{R\tau _\text {max}} \left( n_{\text {th}}+\frac{1}{4R\tau _\text {max}}\right) \right] ^{1/2} \right. \nonumber \\&\qquad \qquad \left. -\frac{1}{2R\tau _\text {max}}\right) \end{aligned}$$

where $\delta f_\text {res} = f_\text {res}(T_\text {bath},T_\text {load})-f_0$, $f_0=f_\text {res}(T_\text {bath}=0,T_\text {load}=0)$, $\alpha =0.24$-$-$0.33 is the kinetic inductance fraction, $\eta _{\textrm{opt}}$ is the total optical efficiency, $\eta _{pb}$ is the pair-breaking efficiency given in Table 1, $T_\text {exc}$ accounts for a fixed additional (“excess”) optical load due to cryostat emission, $V=3224~\upmu \text {m}^3$ is the volume of the KIDs’ inductor, $\Delta _0\simeq 206~\upmu \text {eV}$ is the gap energy at 0 K, $n_\text {th}$ is the thermal quasiparticle density, $\tau _\text {max}=400~\upmu \text {s}$ is the maximum quasiparticle life time [11–13], $R=2\Delta _0^2/[N_0\tau _0(k_\textrm{B} T_\textrm{c})^3]$ is the recombination rate per unit density of quasiparticles, $N_0=1.07\times 10^{29}~\text {J}^{-1}\upmu \text {m}^{-3}$ is the single-spin electron density of states at the Fermi energy level [10], $k_\textrm{B}$ is the Boltzmann constant, $T_\textrm{c}\simeq 1.36~\text {K}$ is aluminum critical temperature, $\tau _0=438~\text {ns}$ is the characteristic electron–phonon interaction time [14, 15], and $\kappa _2$ is defined as:

2 $$\begin{aligned} \kappa _2 = \frac{1}{\pi N_0 \Delta _0} \left[ 1+\sqrt{\frac{2\Delta _0}{\pi k_b T_\text {bath}}}\exp (-\xi )I_0(-\xi )\right] , \end{aligned}$$

with $I_0$ the zeroth-order modified Bessel function of the first kind, $\xi =\hbar \omega /(2k_\textrm{B}T_\text {bath})$, $\hbar$ the reduced Planck constant, and $\omega = 2 \pi f_{\textrm{res}}$. The values of $\alpha$ and $\Delta _0$ used here were obtained for each KID during a previous experiment, we assume $T_c \approx \Delta _0/(1.76 k_\textrm{B})$, and for each frequency band, we use the value of $\Delta \nu _\text {eff.}$ obtained with the FTS measurement. We measured $\alpha$ and $\Delta _0$ previously with no optical load on the devices, we assume $T_c \approx \Delta _0/(1.76 k_\textrm{B})$, and we use the measured values of $\Delta \nu _\text {eff.}$ from Table 1. In addition to $\eta _{\textrm{opt}}$, we also fit for $f_0$ and $T_\text {exc}$. Figure 4 shows a typical dataset and fit and the inferred values of $\eta _{\textrm{opt}}$. The efficiencies are reasonable considering that they include cryostat optical transmission and that the antireflection layer used for the device substrate is tuned for 200–300 GHz. More detailed modeling is underway, as are measurements of the nature of the response of the “dark detectors” (KIDs not connected to antennas).

## Conclusion

**Table 1:** Simulated and measured parameters for the four spectral bands: $\nu _\text {mean}$ is the center frequency of the band, $\Delta \nu _\text {eff.}$ is the effective bandwidth, $\eta _\text {pb}$ the pair-breaking efficiency estimated with $T_c=1.36~\text {K}$ and $\nu _\text {mean}$ measured [15]. FWHM is the full width at half maximum of the hierarchical phased-array antenna central lobe, as measured in Fig. 2d and theoretically calculated by $(c / \nu _\text {mean})/ L_\text {antenna}$, where *c* is the speed of light in vacuum and $L_\text {antenna}$ the size of hierarchical phase-array antenna pixel (6.66 mm for B2 and 3.33 mm for B3, B4, and B5)

Band	$\eta _{\text {pb}}$	Expectation			Measurement		
		$\nu _\text {mean}$ (GHz)	$\Delta \nu _\text {eff.}$ (GHz)	FWHM	$\nu _\text {mean}$ (GHz)	$\Delta \nu _\text {eff.}$ (GHz)	FWHM
2	0.64	151.02	40.26	$17.1^\circ$	157.84	34.11	$17.3^\circ$
3	0.49	219.52	38.59	$23.5^\circ$	224.16	38.10	$22.0^\circ$
4	0.45	282.84	45.60	$18.2^\circ$	280.33	36.33	$17.9^\circ$
5	0.41	346.67	31.12	$14.9^\circ$	345.12	25.96	$16.3^\circ$

We have demonstrated a two-scale hierarchical antenna/band-pass filter bank architecture that has beam patterns and spectral band passes in reasonable agreement with expectations and good optical efficiency. Further modeling, to be presented in a future publication, will endeavor to explain deviations from simple sinc function expectations. We are currently revising the band-pass filter bank design to reduce ripple, add B1 and B6, and integrate it with a three-scale hierarchical antenna. Hierarchical antennas are thus a promising technology for maximizing the use of the focal plane of expensive, large-aperture (30–50 m) millimeter/submillimeter telescopes.

## Footnotes

1 ^4^He/^3^He/^3^He Chase Cryogenics sorption cooler based on a custom cryostat cooled by a Cryomech PT-415 two-stage pulse tube cooler.

2 61 cm $\times$ 61 cm panel of WAVASORB^®^ VHP, immersed in liquid nitrogen for the cold load.
